# A Suggested New Departure in Hospital Administration

**Published:** 1904-12-03

**Authors:** 


### HOSPITAL ADMINISTRATION.

### CONSTRUCTION AND ECONOMICS.

### A SUGGESTED NEW DEPARTURE IN HOSPITAL ADMINISTRATION.

#### II. THE CHAIRMAN-SUPERINTENDENT.—OBJECTIONS.

In a previous article, published last week, we dealt with the advantages which might be held to attach to a new departure in hospital administration, which we termed the chairman-superintendent system. We will now point out some obvious objections.

The disadvantages of the system must be apparent to all who have an intimate knowledge of the daily inner working of a great hospital. It will be observed that the scheme which we foreshadowed in our last article practically ignores the patients, and that there is no plan for the supervision of the admission, refusal, and discharge of patients, nor any resident superintendent in charge of the resident medical staff, which in a large London hospital must consist mainly or entirely of very young men, just qualified, who have everything to learn in the way of administration, and who individually hold office for a period of about six months. In a system which would place the whole control in the hands of a layman to the exclusion of the doctors, it is not surprising to find that the resident medical staff and everything pertaining to the actual treatment of the patients would be left outside the suggested administrative control. It is not possible to place a layman in a position to exercise efficient control over the resident medical staff of a hospital, or to prevent them from making in good faith the multifarious mistakes and errors which must occur, and do occur, in the course of a year's work at a great hospital, where so much has to be left initially to the student who can have only recently qualified, if he possesses a medical qualification at all.

In practice, therefore, the members of the resident medical staff where such a system would prevail must retire from office before it is really possible for them to master efficiently all the duties pertaining to their office, so far as they may affect the economy and discipline of a great hospital. It has been frequently shown in the past that in the case of hospitals where there are resident medical officers who retain office for several years a change in one of these officers may mean, in fact, a large increase in the expenditure on the beds which come immediately under his control during the first year of his office. This is so because he cannot appreciate without experience the directions in which saving may be effected, extravagance avoided, and the persuasive influence of the sister of the ward resisted without injury to the patients. In regard, too, to the admission and discharge of in-patients, especially where these admissions are left largely or entirely in the hands of inexperienced juniors who are endowed with privileges in regard to their rights to treat certain minor operative and other cases, there must be a tendency for the annual returns to show a much greater number of patients passing through the beds than in the case of a hospital where the whole of the admissions are under the direct control of an experienced and able medical superintendent who decides in all doubtful cases as to what course shall be taken before the patient is admitted to a ward or refused admission. In the same way, too, the absence of experience and the keen desire of the junior to admit every case of special interest which comes under his notice may increase the risk of patients being sent out of a hospital more rapidly than is good for their welfare or for the reputation of the hospital itself. After all the true aim and purpose of a great hospital should be first of all to provide the maximum of comfort and security for the patients, and at the same time to secure that no case once admitted shall be allowed to leave the hospital unless steps have been taken to guarantee that the circumstances of the particular patient and his immediate physical needs are supplied by removal to a convalescent home or to his own home, where he should possess everything which may be required to expedite to the fullest extent his complete restoration to health.

Economically, too, the system is not likely to prove successful. A medical superintendent with an accurate knowledge and experience of his work, by continuous inspection of every department of the hospital, is able to see where there is waste, the causes of that waste, and how those at fault can be made responsible. When a resident medical officer has but six months to learn the practical side of his medical or surgical work, he has a great deal too much to attend to, and to learn to have time to pay much attention to the economical aspects of ward administration, much less to prevent waste by others. It follows, and is demonstrably provable, that there must be a large amount of waste and, as a consequence, much preventible expenditure at a hospital where there is an absence of control and supervision by one responsible officer whose duty it is to be continually on the look out for a leakage in each department. Of course, it is absurd to suppose that a chairman-superintendent, however much he might be in telephonic touch with the heads of departments in a great hospital, could exercise any real control over such leakages as we are here considering, which in the aggregate may frequently amount to a very large sum in the course of 12 months. Then, too, the hospital may suffer from the same want of supervision, the results of which the newspapers have frequently recorded in the past by the publication of the many so-called hospital scandals which arise from what are known as drunk or dying cases. In the old days a young resident without experience was inclined to refuse admission to any but the most urgent cases. Nowadays the disposition of the majority of young resident medical officers must be to grow impatient at the very idea of admitting a case unless it offers some special attraction for clinical or operative purposes. Indeed, the operative mania which has grown up, and is still extending in this country, threatens to reduce many of our great hospitals to places from which patients suffering acutely from chronic maladies, who were formerly received as in-patients, will soon be practically excluded altogether. This evil is an increasing one, and if it is not checked may render it impossible for a student to obtain experience in a multitude of cases without which he will certainly be unable to enter upon general practice. The medical superintendent, with the aid of his assistant, checks all these tendencies and takes care to so control the admissions to the wards that they shall be made to serve the best interests of the public, and at the same time to contain the right kind of cases for clinical purposes. In this way it will be seen that the chairman-superintendent system might ultimately tend to destroy much of the public utility of a great hospital.

The effect, especially on the night work of a great hospital, of a want of continuous supervision by a man of experience, but illustrates the tendency such a system would have to lower the general discipline of the whole hospital in the absence of any residential chief in supreme authority. It presents the further danger of encouraging the head of what might be a relatively minor department, providing such an official possessed more brains and ambition than the other residents, to gradually gain control of other departments, or to interfere with them by reports or criticism. So friction must exist, however much it might be suppressed, and would thus tend to inefficiency at the bottom, however smooth the working might appear to be to the casual observer. Again, the world being what it is, and a great hospital is after all a little world of its own, such a system must ultimately tend to produce a conscious or semi-conscious competition for the favour of the all-powerful chairman-superintendent, to whom, unless he was a man of surprising strength, more and more gossip would be likely to be brought, and so indiscipline and ill-feeling must be created. We as a nation have seen the dangers and difficulties of conducting war and government throughout the Empire when those on the spot are liable to receive instructions by cable from the home authorities, who for political or other reasons may interfere at a critical stage in the campaign, though the country would often be much better served were no such interference possible. We believe it to be highly probable that a chairman superintendent in telephonic touch with the head of every department of a hospital must necessarily weaken the internal control everywhere, unconsciously it may be, by taking the heart out of the men and women in charge of the work, who have always to maintain order and discipline amongst the staff. This state of uncertainty and general weakening of control must be emphasised when the time came for a chairman-superintendent to resign his post, owing to the difficulty there would be to replace him. His successor too would be largely at the mercy of individual members of the resident staff who from experience would have learnt to so organise their departments as to keep them well under their own control, with the natural wish to cause them to come as little as possible under the notice of the chairman.

Speaking from experience, we incline to think that the idea of telephonic touch with every department of a great hospital, by a man much occupied with his own business, must, in practice, present many danger and risks, have a weakening effect upon the morale of the individual officers, and possibly degenerate into a dangerous absurdity. On the other hand, a chairman or treasurer of a hospital who is the responsible head of the managing body, who confines himself to the exercise of control over the administration as a whole, becomes in practice a tower of strength to a great hospital. Where, however, any chairman or treasurer, even should he devote two days in each week to the work, is tempted to go outside his proper functions and so endeavours from the highest motives, to keep in touch with all the executive details of each department of the entire hospital, the result must ultimately prove unsatisfactory. It is a good rule that every man should stick to his last, and that in the case of hospitals the governing body should have the wisdom to pay adequate salaries and to so organise their system as to have complete supervision and control everywhere. Such perfect administrative control is only possible in the case of great hospitals where an able medical superintendent is responsible for the whole of the details, with the assistance of a resident staff of the highest attainments. Next week we propose to show why and how it is necessary for every great hospital in these days to be under the immediate and continuous control of a medical superintendent of proved experience and technical knowledge.

